# Effects of Agricultural Management Policies on the Exposure of Black-Winged Stilts (*Himantopus himantopus*) Chicks to Cholinesterase-Inhibiting Pesticides in Rice Fields

**DOI:** 10.1371/journal.pone.0126738

**Published:** 2015-05-13

**Authors:** Gregorio M. Toral, Riad E. Baouab, Mónica Martinez-Haro, Inés S. Sánchez-Barbudo, Juli Broggi, Josue Martínez-de la Puente, Duarte Viana, Rafael Mateo, Jordi Figuerola

**Affiliations:** 1 Department of Wetland Ecology, Estación Biológica de Doñana, CSIC, Sevilla, Spain; 2 Université Mohammed V, Rabat, Morocco; 3 Departamento de Sanidad Animal, IREC, CSIC, Ciudad Real, Spain; Ghent University, BELGIUM

## Abstract

Levels of exposure to pesticides in rice fields can be significant depending on the environmental policies practiced. The aim of European Union integrated management policy is to reduce pesticide use and impact on environment. Rice fields provide an alternative breeding habitat for many waterbirds that are exposed to the pesticides used and therefore can be valuable indicators of their risk for wildlife. To evaluate integrated management success we examined exposure of Black-winged Stilts (*Himantopus himantopus*) to cholinesterase-inhibiting pesticides in rice fields under different types of management by measuring plasma cholinesterase activity. Cholinesterase activity was lower in birds sampled in (a) 2008 after a period of intense pesticide application, than in (b) 2005-2007 and 2011 in rice fields subject to integrated management in Doñana (SW Spain) and (c) in control natural wetlands in Spain and Morocco. During 2009 and 2010, cholinesterase activity was lower in rice fields in Doñana than in rice fields in Larache and Sidi Allal Tazi (NW Morocco). Our results suggest that integrated management successfully reduced the exposure of Black-winged Stilts to pesticides in most of the years. Care should be taken to implement mosquito and pest crop controls on time and with environmentally friendly products in order to reduce its impact on wildlife.

## Introduction

Rice fields are used by a variety of waterbirds as breeding and foraging sites [1–3]. The importance of this crop as an alternative habitat for waterbirds has been previously reported (e.g. [3–6]). However, waterbirds using rice fields may be exposed to potentially toxic chemical compounds [7], and only few studies have ever investigated the exposure of rice field-associated fauna to pesticides [8]. Several pesticides used extensively in rice cultivation are extremely toxic to birds and are thought to cause frequent and largely unavoidable mortality [8]. Among the insecticides most commonly used in rice fields are cholinesterase (ChE)-inhibiting agents such as organophosphates (OP) and carbamates (CB). Smith [9] suggested that over 50% of OPs and over 90% of CBs are ‘extremely toxic’ to most bird species. ChE-inhibiting pesticides are the most common insecticides used in industrialized nations, but also in developing countries where their use is generally poorly regulated [10].

ChE is an important enzyme in the central and peripheral nervous systems and is responsible for the hydrolysis of the neurotransmitter acetylcholine (ACh) at the nerve—nerve or nerve—effector interface. Without hydrolysis, ACh accumulates in the synapse, thereby disrupting neurotransmission, impairing behaviour and physiology, and eventually leading to death [11]. Plasma ChE activity can reveal exposure to pesticides that is consistent with intoxication and death in subsets of a population [12], as well as the lack of such exposure [13]. Although ChE-inhibiting pesticides degrade quite rapidly, they can be highly toxic in the short term and may pose a risk to non-target species [8, 14]. For example, shorebird mortality has been reported in rice fields after the application of carbofuran, a potent anti-ChE agent (e.g. [15]). In the rice fields of the Ebro delta (NE Spain), the massive use of OP and CB pesticides (involving more than 20 000 t/yr) caused several waterbird mortality events [16]. In addition to direct mortality, exposure to OP and CB compounds also has sublethal effects [14]. Physiological and behavioural effects such as decreased food intake, hypothermia and altered concentrations of reproductive hormones, which have a great potential for reducing the productivity and survival of free-living wildlife, have been associated with sublethal reductions in brain ChE activity [17]. The repeated application of pesticides in rice fields may also affect the quality (diversity, size) and quantity (density, biomass) of many of the invertebrates that constitute the diet of many waterbirds in this environment [18]. Measuring plasma ChE enzyme activity is a widely used non-destructive method of monitoring avian exposure to ChE-inhibiting pesticides [14, 19–21].

Despite the existence of a few studies on the negative effects of pesticides applied in rice fields on waterbirds (e.g. [14–16, 22], little attention has been paid to the possible attenuation of these effects through management policies. The basis of integrated management are reducing the use of pesticide by using non-chemical treatments when available and rely on the use of a reduced number of low toxicity pesticides when pest abundance increases over threshold established from economic considerations [23]. Although the link between organic and integrated farming, and the reduction of chemical residues in food is widely acknowledged [24], little is known about the presumed positive effects integrated management may have on other environmental components, particularly on wildlife [25]. In southern Europe and North Africa large areas of wetlands have been transformed into rice fields. In Europe, where rice fields cover 581 978 ha [26], changes in the European Union’s Common Agriculture Policy could alter pesticide use and thus its effects on waterbirds. In addition, rice fields in North Africa are subject to different pesticide regulations and rates of use, facts that may alter the way in which these chemicals affect waterbirds.

In this paper we examine the exposure of Black-winged Stilt (*Himantopus himantopus*) chicks to pesticides in rice fields and explore whether differences in pesticide management techniques translate into differences in their negative effects on birds. It is expected that in rice fields in which management puts limits on pesticide application (integrated management) waterbirds will be less affected by pesticides. Further, in developing countries like Morocco, where there are fewer controls on pesticide application, waterbirds breeding in rice fields are liable to be more affected by pesticides. We examined plasma ChE activity, a non-destructive biomarker, in chicks from rice fields in Doñana (SW Spain) cultivated from 2005 to 2011 using two different pesticide management regimes (integrated and intensive). In addition, during 2009–2011 we compared ChE activity in birds from rice fields in Doñana with those in birds from NW Morocco.

## Materials and Methods

### Study sites

**Fig 1:**
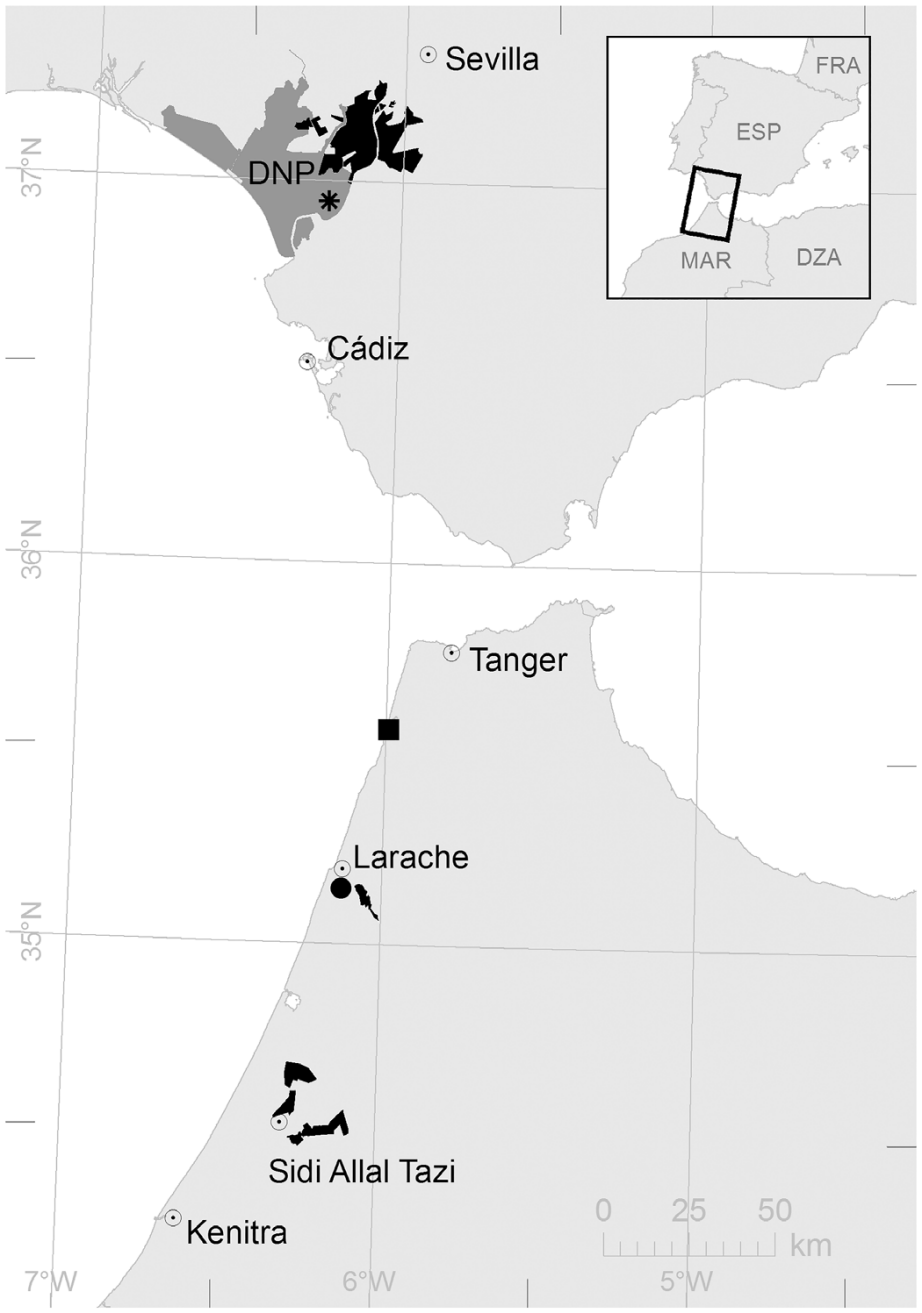
Study sites at Doñana (Spain), Sidi Allal Tazi and Larache (Morocco). Rice fields are shown in black and protected area in Doñana (DNP) is shown in grey. The natural areas are identified with symbols: Doñana (asterisk), Briech (square) and Larache (circle).

Black-winged Stilt chicks were captured during the breeding season (from April to August) from 2005–2011 in Doñana rice fields located near the Guadalquivir marshes (37°09N 06°08W, SW Spain), in 2009–2010 in rice fields of Sidi Allal Tazi and in 2009–2011 in rice fields of Larache (both sites in NW Morocco, 34°30N 6°16W and 35°11N 6°07W respectively). Birds were also sampled at natural ponds in the Doñana area in 2010–2011 (37°04N 6°27W), and in natural wetlands at Larache and a coastal lagoon in Briech (NW Morocco, 35°11N 6°07W and 35°31N 6°00W respectively) in 2011 (Fig 1).

Doñana rice fields constitute the largest cultivated rice area in Spain (36 000 ha), and lie within the marshes of Doñana Natural Space a protected area of about 108 087 ha at the Guadalquivir river delta. The Doñana wetlands (about 27 000 ha) are located within the Guadalquivir marshes and are one of the most important habitats for migratory waterbirds in the Western Palaearctic [27]. The studied rice fields in north western Morocco (about 13 000 ha) are located in sub-humid zones in the regions of Kenitra (Oued Sebou) and Larache (Oued Loukkos) (Fig 1).

### Application of ChE-inhibiting pesticides

During the crop seasons of 2005–2007 and 2009–2011, the Doñana rice fields were cultivated using integrated management, which is characterized by the limited (although not strictly forbidden) use of chemicals (fertilisers and pesticides), and the implementation of production systems with low environmental impact. Integrated management of rice fields in Doñana started in 10.000 Ha by 1998 [28], but the surface has increased since them and the list of authorised pesticides has also changed. In Spain, the main set of norms regarding integrated management derives from the application of Council Regulations (EEC) N°. 2078/92 and subsequently N°. 1257/99. The pesticides and herbicides used in rice fields of Doñana during the study period included mainly malathion, trichlorfon, copper sulphate, propanil and molinate, as well as various other compounds in smaller amounts (as stated by Aparicio et al. [28] and Annual Reglaments of Integrated Management). In 2007, the European Commission removed the OPs malathion and trichlorfon from the list of active substances authorized under Directive 91/414/CEE (decisions 2007/389/CE and 2007/357/CE). These insecticides were still applied in the Doñana rice fields during 2007 under the integrated management policies (hereafter integrated management A, spanning 2005–2007); however, during 2009 two new insecticides were used in the rice fields (integrated management B, spanning 2009–2011): etofenprox (pyrethroid ether) and imidacloprid (neonicotinoid) as alternatives to malathion and trichlorfon. During 2008 crops were intensively managed (hereafter 'intensive management'), that is, farmers used preventive pesticide use with less restrictions in their composition than under integrated management. We had no information about the pesticides applied in Doñana during 2008 due to a lack of reports from farmers.

The two areas of rice fields studied in Morocco are managed differently. In Sidi Allal Tazi rice is cultivated in groups of small paddies managed by different farmers and pesticides are applied by hand. The biological control of algae and mosquito larvae relies mostly on the introduction of the Mosquitofish (*Gambusia affinis*). On the other hand, in Larache rice is cultivated more intensively in large paddy fields. Mosquitoes represent a serious nuisance for the human population in the area, and aerial spraying of pesticides takes place. We lack information regarding the pesticides used in these Moroccan rice fields.

### Avian sampling

Black-winged stilts chicks were captured at night on the banks between paddies and inside the rice paddies using a torch and a dip net. Birds were individually marked with a metallic ring and with a white PVC ring with a three-digit code that allowed birds to be identified subsequently with a telescope. Body mass was measured on a portable digital balance (accuracy 0.1 g). Measurements of the right tarsi length were taken using a digital caliper to the nearest 0.01 mm. Up to 0.3 ml of blood was collected from the jugular vein with 0.5 ml syringes. Blood was stored in eppendorf tubes with heparin, and transported to the laboratory in coolers before centrifugation within 6 h. Plasma was then separated and frozen at -80°C until analysed. Capture and bleeding of birds for this study was done with permits issued by Junta de Andalucia in Spain and from the Institute Scientifique du Rabat and Institute Agronomique in Morocco. The sampling protocols in both countries were approved by the Institutional Animal Care and Use Committee from CSIC.

### Plasma ChE activity

The measurement of ChE activity in plasma samples (10 μl) was performed colorimetrically with a spectrophotometer Ultrospec 2100 pro, UV/Visible, Amersham Biosciences, according to the modification of Ellman´s method described in Martínez-Haro et al. [19]. Cholinesterase hydrolyzes acetylthiocholine iodide (ASChI) into thiocholine and acetate. Thiocholine reacts with dithiodinitrobenzoic acid (DNTB) to form thionitrobenzoic acid, which has a yellow colour that can be measured at 405 nm. The rate of colour production represents ChE activity. Chemical and reagents were Trizma base (tris [hydroxymethyl] aminomethane reagent grade, minimum 99.9%) and Trizma hydrochloride (tris [hydroxymethyl] aminomethane hydrochloride reagent grade) from Sigma; DTNB (5,5´-dithiobis (2-nitrobenzoic acid, 99%)) and ASChI (s-acetylthiocholine iodide 98%) from Alfa-Aesar. Plasma ChE activities were given in mU/ml after the product of ΔA/min with 11 700 [29]. All samples were analysed in the same laboratory but in three different periods. Samples from 2005 to 2008 were analysed in one batch in 2008, samples from 2009 were analysed in 2009 and samples from 2010 and 2011 were analysed in 2011. All the analyses were done with recently prepared reagents and the absorbance of blanks was also measured. Nine replicates of control sample of human serum (Biochemistry Level I, BioSystems) were analysed and the obtained result of cholinesterase activity (mean: 6,597, SD: 359 mU/ml, CV: 5.4%) were within the expected values of the sample (mean: 6300, range: 4725–7875 mU/ml). Duplicate analyses of 16 samples of different years at the end of the study showed the high inter-assay repeatability of the analyses and the stability of the frozen samples at -80°C (Repetability = 0.93). The intra-assay variation between five repeated measurements of a single sample was 2.1% (n = 5, mean = 1130, SD = 24 mU/ml).

### Statistical analyses

Mixed Model ANOVA’s were used to explore factors related to ChE of Black-winged Stilts. The models were fitted using JMP 9.0 [30]. First we compared ChE levels across years, rice field areas and control areas from Spain and Morocco. Only one individual was captured in 2006 and was not included in the analyses. Family was included as a random factor identifying samples from siblings. No differences in ChE levels between control areas in Spain and Morocco were found in a preliminary analysis (F_1,18.09_ = 0.15, p = 0.71) and for this reason data from both control areas were merged into a single control group in subsequent analyses. The dataset from Doñana was analyzed with ChE as the dependent variable and ‘pesticide management’, ‘year’ nested inside ‘pesticide management’, and ‘body mass’ and ‘tarsus length’ as independent variables. The variable ‘pesticide management’ was coded as a nominal variable with value 0 for intensive management, 1 for ‘integrated management A’ and 2 for ‘integrated management B’. Family was included as a random factor grouping samples coming from siblings. We included tarsus length and body mass as independent variables to control for a possible body-size effect on ChE. In order to test whether body condition was affected by pesticide exposure, we built another model using ‘body mass’ as dependent variable, with ChE and ‘tarsus length’ as independent variables, and family as a random factor. In a similar way, we analyzed differences in ChE activity between (a) chicks sampled in 2009–2010 from Doñana rice fields and (b) chicks captured in 2009–2010 at rice fields in Morocco. The model included ChE as the dependent variable; ‘locality’ (Doñana rice fields, Larache, Sidi Allal Tazi), ‘year’ and ‘body mass’ and ‘tarsus length’ were used as independent variables. Family was included as a random factor. In order to test whether body condition was affected by pesticide exposure we built another model with ‘body mass’ as the dependent variable, ‘locality’, ‘year’, ChE and ‘tarsus length’ as independent variables, and family as a random factor.

## Results

**Fig 2:**
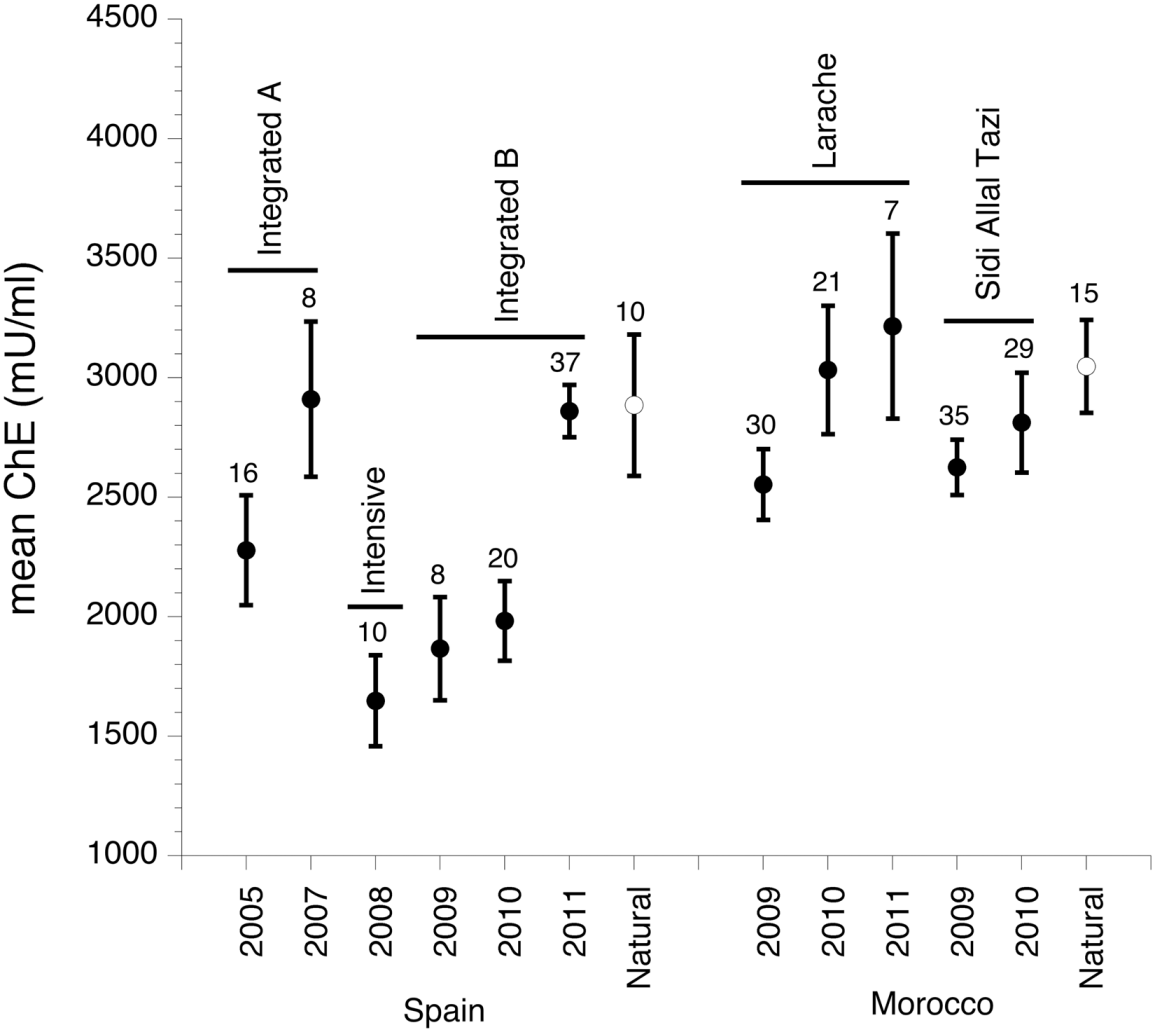
Total cholinesterase activity of Black-winged Stilt (*Himantopus himantopus*) chicks born over the period 2005–2011. Mean ± s.e. total cholinesterase (ChE) activity (mU/ml plasma) and sample size is given over each bar. Filled circles represent data from birds captured in rice fields, and open symbols data from birds captured in natural marshes.

Important differences were found in ChE levels at the different localities and in different years (n = 246, F_11,174.6_ = 4.18, p < 0.0001, Fig 2). In comparison to control areas, ChE values were lower in Doñana rice fields in 2005, 2008–2010 (p < 0.05 in a-posteriori t-tests). No differences in ChE were found in birds sampled in control areas and rice fields in Morocco. In the same way ChE levels in control areas in Doñana and Morocco were similar to those found in Doñana rice fields in 2007 and 2011 (Fig 2). A total of 98 Black-winged Stilts sampled during 2005–2011 were included in the analyses of differences in ChE in relation to pesticide management in the Doñana rice fields. ‘Pesticide management’ was the only variable significantly related to ChE activity (F_2,74.23_ = 4.58; p = 0.01). Plasma ChE activity in 2008 (intensive management) was lower than in 2005–2007 (integrated management A; a-posteriori t test, p < 0.05), and 2009–2011 (integrated management B; p < 0.05) while no differences were detected between the two periods with integrated rice management. During the period of integrated management B, ChE activity in 2009 and 2010 was significantly lower than in 2011 (p < 0.05) (Fig 2). Body mass and tarsus length were unrelated to ChE (body mass: F_1,86.56_ = 0.41, p = 0.52; tarsus length: F_1,89.34_ = 0.40, p = 0.53). Further, body condition was unaffected by pesticide exposure. Only tarsus length was related to body mass (F_1,88.57_ = 330.56, p < 0.0001) while no relationship with ChE activity was found (F_1,83.34_ = 1.25; p = 0.27).

A total of 137 Black-winged Stilts sampled during 2009–2010 were included in the analyses of the differences in ChE between individuals from Doñana and Morocco rice fields. ‘Locality’ was the only significant variable (F_2,70.07_ = 4.05; p = 0.02), with smaller ChE values being found in the integrated management B area in Doñana, than in both rice field areas from Morocco (Fig 2). No interaction between year and locality was found (F_2,67.57_ = 0.47, p = 0.62), and tarsus length and body mass were unrelated to ChE activity (p > 0.47 in both cases). In the model built to examine whether body condition was affected by pesticide exposure, only tarsus length was positively related to body mass (F_1,127.10_ = 181.47, p < 0.0001), while ChE (F_1,35_ = 0.01; p = 0.93), locality (F_2,104.70_ = 1.73, p = 0.18) and the interaction ‘year*locality’ (F_2,96.54_ = 1.63, p = 0.20) were unrelated to body mass.

## Discussion

We found important differences in the levels of ChE activity between Black-winged Stilts chicks reared in different locations or under different pesticide management policies. Previous studies of the effects of organic and integrated farming techniques on the environment have demonstrated their positive effects on birds (e.g. [31–33]), although usually only in terms of species richness and abundance.

Exposure to ChE-inhibiting compounds in birds may occur in several ways, including the consumption of contaminated insects and seed, drift or contact with pesticide-coated vegetation and the direct consumption of pesticide granules [34]. However, birds normally receive sublethal doses, primarily by ingesting pesticide-contaminated food items [35]. Reductions in brain ChE activity larger than 50% can be considered evidence for lethal exposure [36]. However, blood or plasma ChE is more sensitive than brain ChE and some difficulties have been found to establish a benchmark as for brain ChE [37]. Several authors consider that birds with plasma ChE activity 2 standard deviations below the mean reference value could be considered evidence for exposure to ChE-inhibiting pesticides [38]. The mean value of ChE in the natural areas was 2983.03±817.90 mU/ml (Fig 2) and 13 individuals did not reach the benchmark of 1349.23 mU/ml. All these 13 individuals were captured in rice-field areas (2 in Larache, 1 in Sidi Allal Tazir and 10 in Doñana, 5 in integrated management B, 2 in integrated management A and 3 in intensive management).

ChE levels in birds captured in control natural areas were higher than in birds captured in intensive farmed fields. Also the higher activity of ChE found in birds from the Doñana rice fields under integrated management as opposed to intensive management confirms our hypothesis that appropriate pesticide management could attenuate the negative effects on birds of these chemical treatments. Interestingly, and contrary to our initial expectative, in 2009 and 2010 we found lower values for ChE in birds from Spain than from Morocco, which may reflect the difference in the intensification of rice production between these two countries. Rice cultivation in Spain is more mechanized and pesticide treatment is carried out on a larger scale than in Morocco, where the use of intensive agriculture is more restricted. Additionally, we can not exclude that pesticide treatments in Morocco had relied on non ChE depressing pesticides. During 2008, 30% of the sampled individuals did not reach the ChE concentration benchmark, while this number ranged between 0 and 6.7% for all the rice field areas studied in Morocco. The proportion of individuals below ChE concentration benchmark in Doñana rice field under integrated management ranged between 0% in 2007 and 2011 and the 20% in 2010.

The low ChE activity values in Doñana in 2009 and 2010 were unexpected given that malathion and trichlorfon had been banned in 2007 and these compounds do not persist in the environment. The banning of these substances produced a lot of complains among farmers. Therefore, we can not exclude that the lower ChE activities in 2008 and the two following years were due to the use of stocks of ChE-inhibiting pesticides. We are not aware of any additional changes in programme implementation, control or farmer formation that may have contributed to these differences. Management actions may need to be evaluated at mid-term periods because cultural inertia and economic factors linked to stock management may limit the success of those measures on the short term and may also explain the intermediate ChE values in 2009–2010 in comparison to 2008 on one side and 2005–2007 and 2011 by the other.

After the ban of malathion and trichlorfon, other non-ChE inhibiting insecticides such as etofenprox and imidacloprid have been included in the integrated management. To the best of our knowledge no studies of the effects of etofenprox on birds have been conducted, and only a few studies on the toxicity of imidacloprid on birds are available [39–40]. In fact, imidacloprid could even be more toxic for birds than previously applied pesticides such as malathion, as has been observed in a recent review of the negative effects on birds of pesticides applied in rice fields [8].

There is a lack of any integrated approach to the evaluation of the overall direct and indirect effects on wildlife of the use of pesticides in rice farming [16]. Proposed management practices that address adverse effects of pesticide use in rice fields include the increased adoption of integrated management principles and the use of less toxic products [8]. Understanding the effects of different farming practices is a high-priority research question that will have consequences for policymaking [41]. Our results suggest that agricultural management policies can have a severe impact on health and condition in breeding birds, and that legislation on pesticide use and agro-environmental schemes can attenuate their negative effects on waterbirds. However, these birds are characterized by their high dispersal capacity [42], as exemplified by the observation of the same Black-winged Stilt breeding in a rice field in Morocco in 2008 and in Doñana in 2010 (personal observation). Therefore, pesticide management at trans-national and continental scales is necessary if we are to reduce exposure to pesticides in waterbirds during their life-cycles and along their migratory pathways.

## Conclusions

Results presented in this study suggest that pesticide management policies influence the way in which rice cultivation affects waterbirds. Birds from the Doñana rice fields presented lower values of ChE when the crop was under intensive management as opposed to the integrated management with more controlled use of pesticides. The reduction in the use of pesticides associated to integrated management may benefit birds, although more studies are necessary to evaluate the negative effects of etofenprox and imidacloprid on waterbirds feeding on rice fields after the ban of ChE-inhibiting insecticides. Moreover, the higher ChE activity found in birds from Morocco may reflect a lower use of ChE-inhibiting pesticides than in the Spanish rice fields under the intensive management and even in some years of integrated management.

## References

[1] Baouab RE. Composition avifaunistique et fonctionnement des rizières de la province de Sidi Kacem (Maroc). Bulletin de l’Institut Scientifique, Rabat, section Sciences de la Vie 2008;30: 37–44. 10.1007/s00402-009-0884-y 19440728

[2] Czech HA, Parsons KC. Agricultural wetlands and waterbirds: a review. Waterbirds 2002;25 (Special Publication 2): 56–65.

[3] Fasola M, Ruiz X. The value of rice fields as substitutes for natural wetlands for waterbirds in the Mediterranean region. Colonial Waterbirds 1996;19: 122–128.

[4] Elphick CS, Oring LW. Conservation implications of flooding rice fields on winter waterbird communities. Agric Ecosyst Environ. 2003;94: 17–29.

[5] Toral GM, Figuerola J. Unraveling the importance of rice fields for waterbird populations in Europe. Biodivers Conserv. 2010;19: 3459–3469.

[6] Tourenq C, Benhamou S, Sadoul N, Sandoz A, Mesleard F, Martin J-L, Hafner H. Spatial relationships between tree-nesting heron colonies and rice fields in the Camargue, France. Auk 2004;121: 192–202.

[7] Lawler SP. Rice fields as temporary wetlands: a review. Isr J Zool. 2001;47: 513–528.

[8] Parsons KC, Mineau P, Renfrew RB. Effects of pesticide use in rice fields on birds. Waterbirds 2010;33 (Special publication 1): 193–218.

[9] Smith GJ. Pesticide use and toxicology in relation to wildlife: organophosphorus and carbamate compounds. US Fish and Wildlife Service, Resource Publication 170; 1987.

[10] Pimentel D, Levitan L. Pesticides: amounts applied and amounts reaching pests In: Pimentel D, editor. CRC handbook of pest management in agriculture. Boca Raton: CRC Press; 1991 pp. 741–750.

[11] Grue CE, Gibert PL, Seeley ME. Neurophysiological and behavioral changes in non-target wildlife exposed to organophosphate and carbamate pesticides: thermoregulation, food consumption, and reproduction. Am Zool. 1997;37: 369–388.

[12] Hooper MJ, Detrich PJ, Weisskopf CP, Wilson BW. Organophosphorus insecticide exposure in hawks inhabiting orchards during winter dormant-spraying. Bull Environ Contam Toxicol. 1989;42: 651–659. 2742994 10.1007/BF01700383

[13] Goldstein MI, Lacher TE Jr, Zaccagnini ME, Parker ML, Hooper MJ. Monitoring and assessment of Swainson's Hawks in Argentina following restrictions on monocrotophos use, 1996–97. Ecotoxicology 1999;8: 215–224.

[14] Strum KM, Hooper MJ, Johnson KA, Lanctot RB, Zaccagnini ME, Sandercock BK. Exposure of nonbreeding migratory shorebirds to cholinesterase inhibiting contaminants in the western hemisphere. Condor 2010;112: 15–28.

[15] Flickinger EL, Mitchell CE, White DH, Kolbe EJ. Bird poisoning from misuse of the carbamate Furadan in a Texas rice field. Wildl Soc Bull. 1986;14: 59–62.

[16] Mañosa S, Mateo R, Guitart R. A review of the effects of agricultural and industrial contamination on the Ebro Delta biota and wildlife. Environ Monit Assess. 2001;71: 187–205. 11686200 10.1023/a:1017545932219

[17] Grue CE, Hart ADM, Mineau P. Biological consequences of depressed brain cholinesterase activity in wildlife In: Mineau P, editor. Cholinesterase-inhibiting insecticides, Chemicals in Agriculture Vol. 2 Amsterdam: Elsevier; 1991 pp. 151–209.

[18] Tourenq C, Sadoul N, Beck N, Mesléard F, Martin JL. Effects of cropping practices on the use of rice fields by waterbirds in the Camargue, France. Agric Ecosyst Environ. 2003;95: 543–549.

[19] Martínez-Haro M, Viñuela J, Mateo R. Exposure of birds to cholinesterase-inhibiting pesticides following a forest application for tick control. Environ Toxicol Pharmacol. 2007;23: 347–349. 10.1016/j.etap.2006.11.011 21783779

[20] McInnes PF, Andersen DE, Hoff DJ, Hooper MJ, Kinkel LL. Monitoring exposure of nestling songbirds to agricultural application of organophosphorus insecticide using cholinesterase activity. Environ Toxicol Chem. 1996;15: 544–552.

[21] Parsons KC, Matz AC, Hooper MJ, Pokras MA. Monitoring wading bird exposure to agricultural chemicals using serum cholinesterase activity. Environ Toxicol Chem. 2000;19: 1317–1323.

[22] Strum KM, Alfaro M, Haase B, Hooper MJ, Johnson KA, Lanctot RB. Plasma cholinesterases for monitoring pesticide exposure in Nearctic-Neotropical migratory shorebirds. Ornitol Neotrop. 2008;19: 641–651.

[23] Kogan M. Integrated Pest Management: Historical perspectives and contemporary developments. Annu Rev Entomol. 1998;43: 243–270. 9444752 10.1146/annurev.ento.43.1.243

[24] Woese KD, Lange C, Bogl KW. A comparison of organically and conventionally grown foods—results of a review of the relevant literature. J Sci Food Agric. 1997;74: 281–293.

[25] Dempster JP. Effects of pesticides on wildlife and priorities in future studies In: Brent KJ, Atkin RK, editors. Rational pesticide use. Cambridge: Cambridge University Press; 1987 pp. 17–25.

[26] Ferrero A, Nguyen N. Constraints and opportunities for sustainable development of rice-based production systems in Europe Paper presented at the International Conference on Sustainable Rice Systems, 12–13 2 2004, FAO, Rome, Italy.

[27] Rendón MA, Green AJ, Aquilera E, Almaraz P. Status, distribution and long-term changes in the waterbird community wintering in Doñana, south-west Spain. Biol Conserv 2008;141: 1371–1388.

[28] Aparicio S, Jurado N, Montes D, Carrascal JF, Cano M. Memoria general: campaña de producción integrada de arroz en la provincial de Sevilla Año 2007. Sevilla: Federación de Arroceros de Sevilla; 2008.

[29] Hill EF, Fleming WJ. Anticholinesterase poisoning of birds: field monitoring and diagnosis of acute poisoning. Environ Toxicol Chem 1982;1: 27–38.

[30] SAS Institute Inc. Using JMP 9. Cary: SAS Institute Inc; 2010.

[31] Beecher NA, Johnson RJ, Brandle JR, Case RM, Young LJ. Agroecology of birds in organic and nonorganic farmland. Conserv Biol 2002;16: 1620–1631.

[32] Freemark KE, Kirk DA. Birds on organic and conventional farms in Ontario: partitioning effects of habitat and practices on species composition and abundance. Biol Conserv 2001;101: 337–350.

[33] Genghini M, Gellini S, Gustin M. Organic and integrated agriculture: the effects on bird communities in orchard farms in northern Italy. Biodivers Conserv 2006;15: 3077–3094.

[34] Gard N, Hooper M. An assessment of potential hazards of pesticides and environmental contaminants In: Martin T, Finch D, editors. Ecology and Management of Neotropical Migratory Birds. New York: Oxford UniversityPress; 1995 pp. 294–310.

[35] Matsumura F. Toxicology of insecticides. New York: Plenum; 1975.

[36] Ludke JL, Hill EF, Dieter MP. Cholinesterase (ChE) response and related mortality among birds feed ChE inhibitors. Arch Environ Contam Toxicol 1975;3: 1–21. 1130829 10.1007/BF02221128

[37] Glaser LC. Organophosphorus and carbamate pesticides, In: Fiend M, Franson JC, editors. Field Manual of Wildlife Diseases. Madison: USGS; 1999 pp 287–293.

[38] Maul JD, Farris JL. Monitoring exposure of Northern cardinals, *Cardinalis cardinalis*, to cholinesterase-inhibiting pesticides: enzyme activity, reactivations, and indicators of environmental stress. Environ Toxicol Chem 2005;24: 1721–1730. 16050589 10.1897/04-385r.1

[39] Berny P, Buronfosse F, Videmann B, Buronfosse T. Evaluation of the toxicity of Imidacloprid in wild birds: a new high performance thin layer chromatography (HPTLC) method for the analysis of liver and crop samples in suspected poisoning cases. J Liq Chromatogr. 1999;22: 1547–1559.

[40] López-Antia A, Ortiz-Santaliestra ME, Mougeot F, Mateo R. Experimental exposure of red-legged partridges (Alectoris rufa) to seeds coated with imidacloprid, thiram and difenoconazole. Ecotoxicology 2013;22: 125–138. 10.1007/s10646-012-1009-x 23111803

[41] Sutherland WJ, Armstrong-Brown S, Armsworth PR, Tom B, Brickland J, Campbell CD, et al The identification of 100 ecological questions of high policy relevance in the UK. J Appl Ecol 2006;43: 617–627.

[42] Figuerola J. Climate and dispersal: Black-winged Stilts disperse further in dry springs. PLoS ONE 2007;2: e539 17579713 10.1371/journal.pone.0000539 PMC1891090

